# Cerebral Cavernous Angioma Associated with Klippel Trenaunay Syndrome Treated with Gamma Knife Radiosurgery: Case Report and Literature Review

**DOI:** 10.7759/cureus.4318

**Published:** 2019-03-25

**Authors:** Muhammed Abid Saleem, Noor E Zahra, Fatima Hemani, Abdullah Jan Ali, Aamir Gilani

**Affiliations:** 1 Neurosurgery, Neurospinal and Cancer Care Institute, Karachi, PAK; 2 Neurosurgery, Jinnah Postgraduate Medical Center, Karachi, PAK; 3 Neurosurgery, Jinnah Postgraduate Medical Centre, Karachi, PAK; 4 Internal Medicine, Jinnah Postgraduate Medical Centre, Karachi, PAK

**Keywords:** cerebral cavernous angioma, kts, klippel-trenaunay syndrome, stereotactic gama knife radiosurgery, rap1a, rasa1, krit1, gamma knife

## Abstract

Klippel Trenaunay syndrome (KTS) is a rare, sporadic congenital syndrome characterized by cutaneous hemangiomas, venous varicosities, and osseous-soft tissue hypertrophy of the affected limb. It is genetically heterogeneous, and its clinical presentation is variable.

We report the case of a 13-year-old male with KTS, who presented with a cerebral cavernous angioma in the corpus callosum. To the best of our knowledge, this is the first reported case of its kind from Pakistan and the only known case in the literature of KTS to be treated with stereotactic radiosurgery.

The possibilities of shared genetic pathways between KTS and cavernous angiomas and the need for neurovascular scrutiny in patients with this syndrome are discussed.

## Introduction

Klippel Trenaunay syndrome (KTS, OMIM 149000) is a rare congenital mesodermal phakomatosis characterized by a triad of 1) capillary malformations, 2) venous malformation or widespread early-onset varicose veins, and 3) osseous or soft tissue hypertrophy of the affected limb [1-2]. The presence of two of the three cardinal features is sufficient to diagnose KTS [3]. Vascular anomalies in KTS are the slow flow type. Klippel-Trenaunay-Weber syndrome (KTWS) with fast flow vascular malformation, such as an arteriovenous fistula, is called Parkes Weber syndrome [4].

The clinical presentation of the syndrome is diverse and depends upon the site of malformation, from patients who have cosmetic concerns to patients with life-threatening conditions, such as deep vein thrombosis (DVT), pulmonary embolism (PE), cellulitis, lymphedema, and internal bleeding from diseased vessels [5].

We describe the only known case in the literature of KTS associated with cavernous angioma to be treated with Gamma knife radiosurgery and draw attention to the evidence of a shared genetic pathway between the two conditions.

## Case presentation

A 13-year-old boy, a diagnosed case of KTS, was referred to our hospital with complaints of vertigo for three months. His previous medical history included an intracranial bleed for which he was hospitalized four years ago. There was no history of surgery or radiotherapy. He was born at term after an uncomplicated pregnancy. His developmental milestones were normal. His family history did not show any precedent occurrence of cavernomas, intracerebral hemorrhage, or KTS.

**Figure 1:**
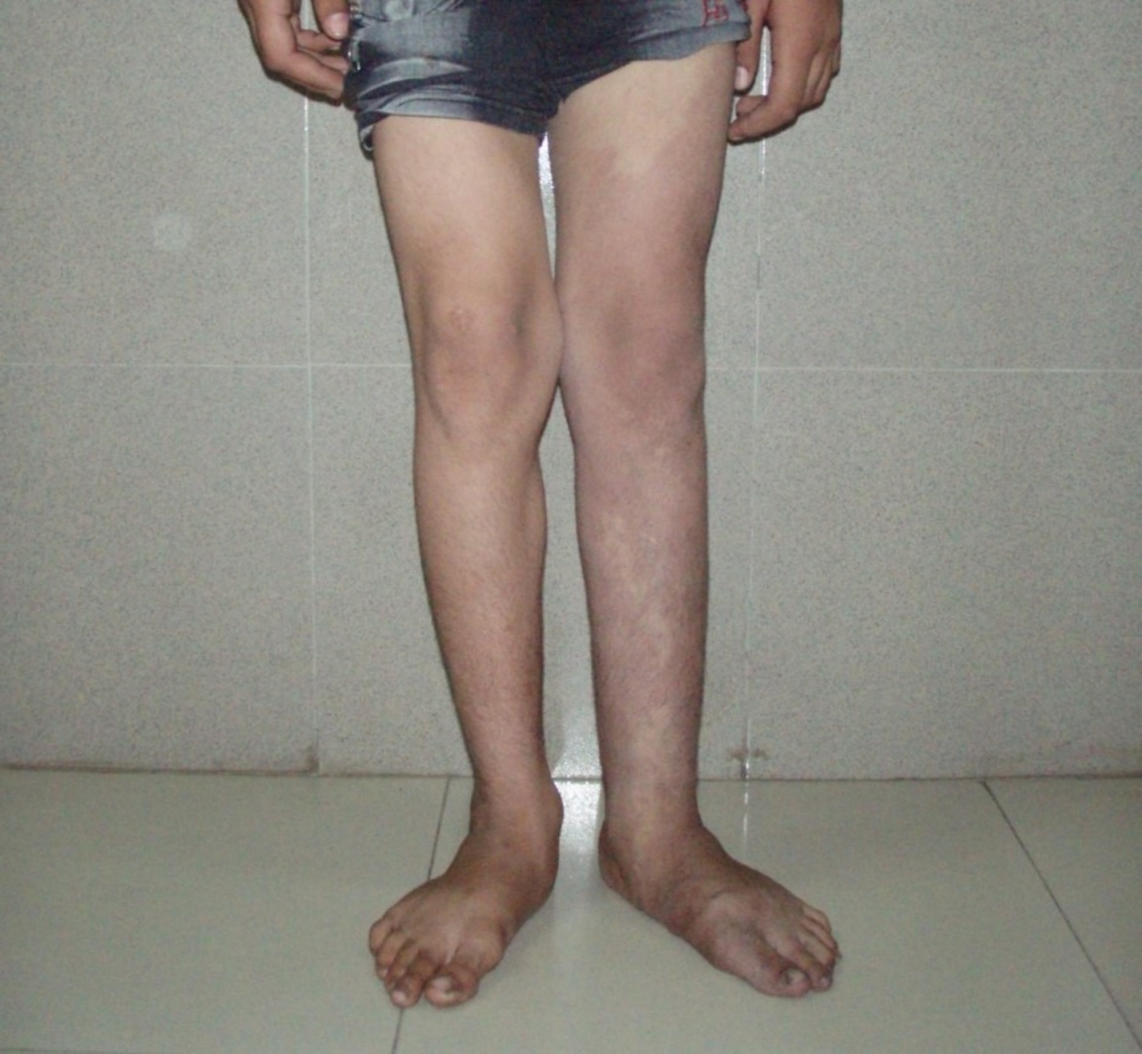
Photograph showing left lower limb hypertrophy, varicosities, and cutaneous angiomatosis.

**Figure 2:**
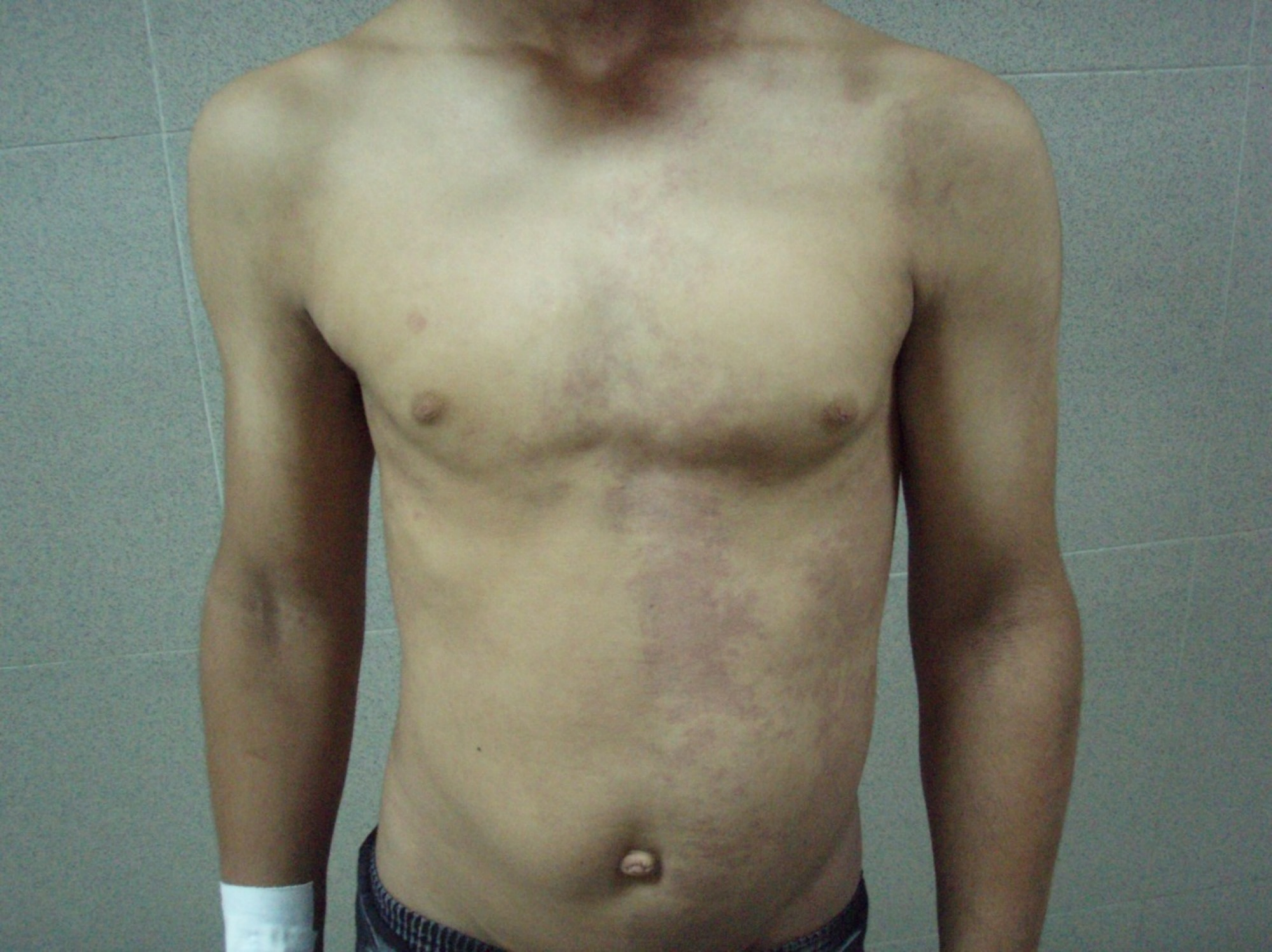
Multiple cutaneous port-wine stains on the left side of the anterior trunk.

**Figure 3:**
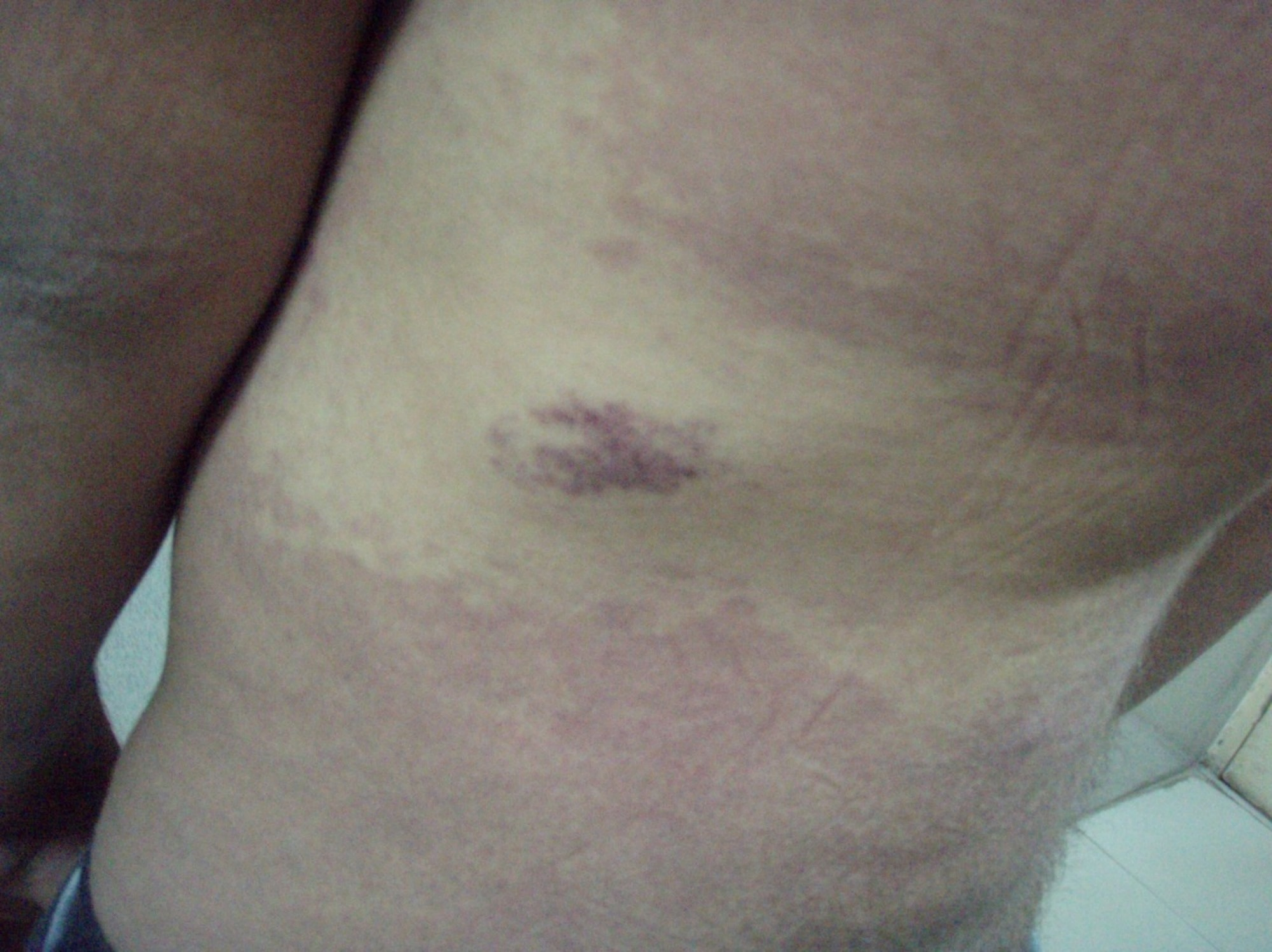
Multiple cutaneous port-wine stains with telangiectasia on the left side of the posterior trunk.

His general physical examination revealed left upper and lower extremity hemihypertrophy and cutaneous angiomatosis of the lower extremities, which were associated with painless varicose veins (Figure 1). Multiple cutaneous port-wine stains with telangiectasia were also observed in the left hand, left anterior chest, and the entire back, which had been evident since birth (Figures 2-3). There was no evidence of syndactyly, polydactyly, congestive cardiac failure, and pulmonary hypertension. No focal neurological deficits were present.

**Figure 4:**
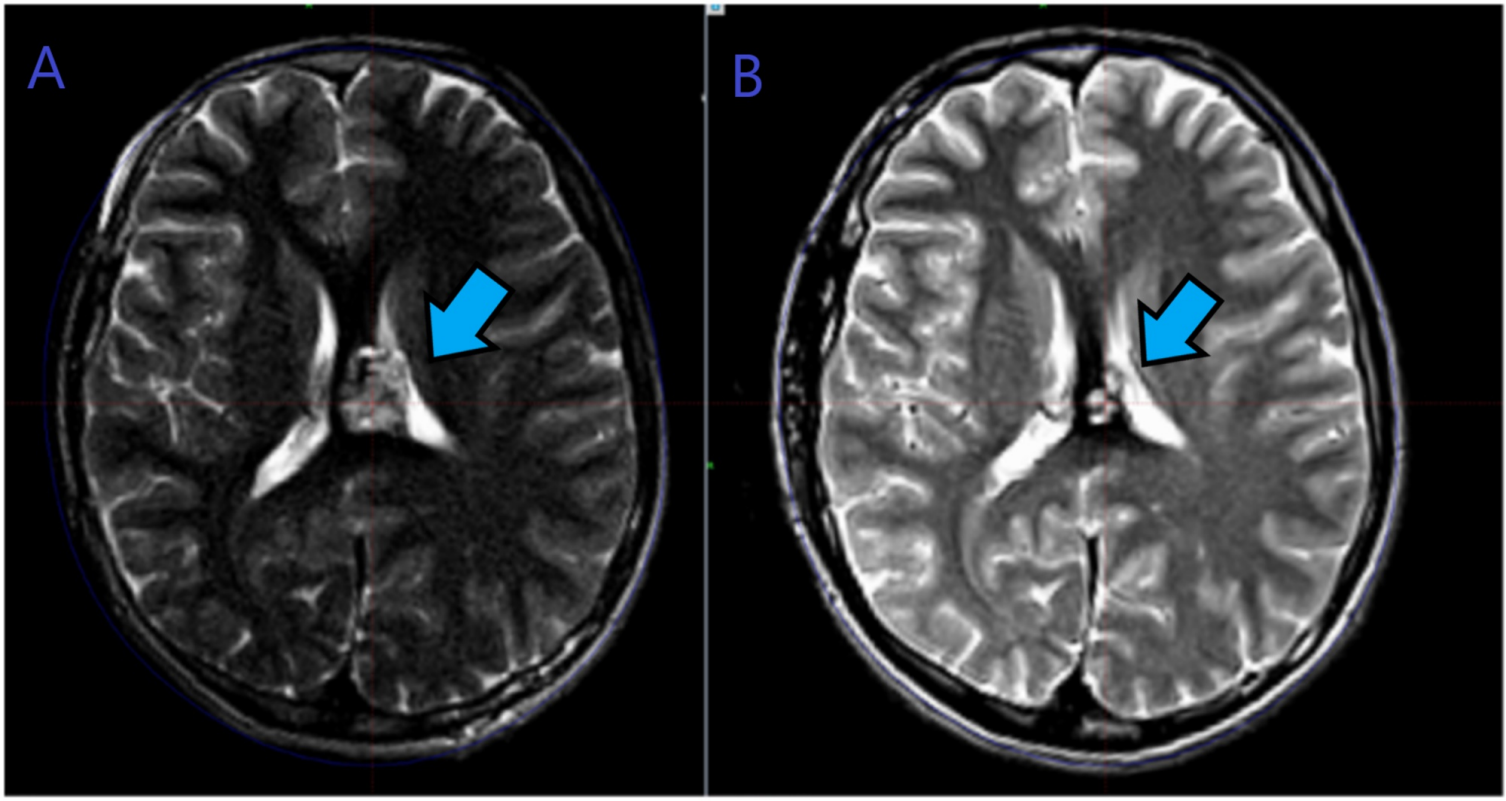
A. MRI brain T2 sequence showing the “pop-corn appearance” of cavernous angioma. Left medial parietal area involving the corpus callosum at the time of Gamma Knife radiosurgery treatment. B. MRI axial cut T2 sequence showing more than 50% regression in the lesion size, six years after the Gamma Knife treatment. MRI: magnetic resonance imaging

Magnetic resonance imaging (MRI) brain, dated August 20, 2009, showed a focal area of abnormal signal intensity noted within the midline involving the medial parietal cortex on the left side as well as the corpus callosum (Figure 4A). After radiographic scans and workup, it was decided to treat this case of a cavernous angioma with Gamma Knife (Elekta, Stockholm, Sweden) radiosurgery using a dose of 16 Gy at 50%, an isodose line to the target volume of 2.4 cm^3^. Gamma Knife model 4C was used to treat this case. The first follow-up contrast MRI done on October 6, 2013, showed that there was a re-demonstration of a focal area of abnormal signal intensity noted within the midline area involving the medial parietal cortex on the left side as well as the corpus callosum. Furthermore, some necrotic changes within the lesion, with perilesional edema, were also noted. On January 12, 2018, the second follow-up MRI revealed a more than 50% reduction in the previously targeted left parietal angioma and no evidence of new hemorrhages, demonstrating good lesion control (Figure 4B).

## Discussion

KTS is a congenital vascular disease characterized by extensive malformations involving capillaries (98%), veins (72%), and lymphatics (11%). Overgrowth of the affected limb is seen in 67% of the patients [3,6].

Common complications in KTS are caused by vascular anomalies in the patients’ extremities, such as ulceration, cellulitis, thrombophlebitis, thrombosis, emboli, hemorrhage, and edema. The most frequent CNS disorder found in KTS is hemimegalencephaly. Various other CNS anomalies have been reported such as spinal and cerebral arteriovenous malformation, orbitofrontal varices, multiple aneurysms, carpel tunnel syndrome, epidural and intradural angiomas, cerebral venous thrombosis, and intracranial hypertension secondary to the inadequacy of superficial cortical veins [4].

Unlike other neurocutaneous disorders, such as tuberous sclerosis and neurofibromatosis, neoplasias are infrequent, with orbital rhabdomyosarcoma, meningiomas, astrocytomas, and limb sarcomas reported most commonly. CNS cavernomas are also rarely associated with KTS and, recently, there have been several reported cases.

In 2006, Pichierri et al. were the first to report an intramedullary cervical cavernoma associated with KTS [7]. In 2010, Boutarbouch et al. reported multiple cavernomas in the spinal cord and brainstem [8]. In 2010, Sudmeyer et al. reported a cavernoma of the inferior olive [9]. In 2014, Ricks et al. reported hemorrhagic cavernous malformations in the medulla oblongata [10]. In 2017, Yoshinaga et al. reported a patient with multiple cerebral and spinal cord cavernomas [11]. This is summarized in Table 1.

**Table 1:** Summary of cases of KTS with CNS cavernomas. KTS: Klippel Trenaunay syndrome; CNS: central nervous system

	YEAR CASE WAS PRESENTED	AGE (YEARS)	SEX	SYMPTOMS	SITE OF CAVERNOMA IN CNS	SITE OF SYMPTOMATIC HEMORRHAGE	TREATMENT
Pichierri et al. [7]	2006	37	F	Sensory deficit of upper limbs	Cervical Spinal Cord	Spinal Cord	Resection
Boutarbouch et al. [8]	2010	55	M	Weakness of lower limbs	Brain and Thoracic Spinal Cord	Spinal Cord	Conservative management
Sudameyer et al. [9]	2011	52	F	Subacute slowly progressive action tremor of the upper limbs	Brain	_	Conservative management
Ricks et al. [10]	2014	74	F	Hemiataxia, headache	Brain	Brain	Conservative management
Yoshinga et al. [11]	2017	64	M	Sensory motor weakness of lower limbs. Vesicorectal disturbance	Brain and Lumbosacral Spine	Brain and Lumbosacral Spine	Conservative management
Our Case	2018	13	M	Vertigo, hemihypertrophy, and cutaneous angiomatosis on left limbs with painless varicose veins	Brain	Brain	Gamma Knife Radiosurgery

KTS most commonly occurs sporadically following a somatic mutation, with a "second hit" model used to explain its etiology [12-13]. However, the disease can affect other family members, suggesting an autosomal dominant inheritance [14]^.^ The activation of AGGF1 and mutations in PIK3CA have been reported to explain the pathogenesis of the syndrome [15]. A mutation in E133K, AGGF1 is a functional mutation that increases angiogenesis by the gain-of-function mechanism [6]. Furthermore, the RASA1 gene mutation was discovered to be involved in the pathogenesis of KTWS [16]. RASA1 codes for p-120-RasGAP, a protein that downregulates the MAPK signaling pathway, which mediates cellular growth, differentiation, and proliferation. It also binds to Rap1a, a member of the RAS superfamily involved in integrin-mediated cellular adhesion [17-18].

Familial cerebral cavernous angiomas are caused by loss-of-function mutations in the KRIT1 gene, which is also a binding partner of Rap1a [19-20]. Since both RASA1 and KRIT1 genes interact with the Rap1a protein, this suggests the possibility of a shared genetic pathway between KTWS and cavernous angiomas. While further studies are needed to establish a strong association between the two conditions, in light of these genetic links and the growing number of cases being reported, patients with KTS may need to undergo screening neurological imaging to rule out a possible cavernoma.

## Conclusions

Our patient, a 13-year-old boy with Klippel-Trenaunay syndrome, who had a history of intracranial bleed that was initially managed conservatively, presented to us with complaints of vertigo. Further investigations revealed a cavernous angioma in the corpus callosum, which was treated with Gamma Knife radiosurgery, and follow-up MRIs showed good lesional control. This is the first reported case of a cavernoma associated with KTS to be treated with radiosurgery.

Taking into account the latest model of genetic development described for KTS and its overlap with the pathogenesis of cavernomas as well as the increasing number of cases being reported, the importance of neurovascular scrutiny in patients with KTS cannot be ignored, as it may help prevent devastating complications such as intracranial bleed.
